# Inflammatory transcriptomic signatures and cell type compositions in inflamed and non-inflamed colonic mucosa of ulcerative colitis

**DOI:** 10.1016/j.gendis.2024.101447

**Published:** 2024-10-30

**Authors:** Eun Mi Song, Jahanzeb Saqib, Yang Hee Joo, Zehra Ramsha, Chang Mo Moon, Sung-Ae Jung, Junil Kim

**Affiliations:** a Departments of Internal Medicine, College of Medicine, Ewha Womans University, Seoul 07804, Republic of Korea; b School of Systems Biomedical Science, Soongsil University, 369 Sangdo-Ro, Dongjak-Gu, Seoul 06978, Republic of Korea

Ulcerative colitis (UC), a subtype of inflammatory bowel disease, arises from disrupted gut homeostasis, primarily due to an aberrant innate immune response to intestinal microbiota and an underlying genetic background.^1^ Recently, complete healing of mucosal inflammation has been suggested as a new therapeutic goal in UC treatment.^2^ However, this goal remains challenging, as approximately 20% of individuals in clinical remission still exhibit active mucosal inflammation.^3^ Understanding the molecular alterations underlying this persistent mucosal inflammation is crucial for advancing UC pathogenesis insights and treatment strategies. While previous studies have explored transcriptomic profiles in patients with inflammatory bowel disease,^4^ consistent gene expression characteristics and enriched pathways, particularly in Asian patients with UC, remain unconfirmed. Our study addresses this gap by employing RNA sequencing (RNAseq) to analyze transcriptomic changes in mucosal biopsy specimens. We compare active (UCA) and macroscopically inactive (UCI) inflammatory areas during colonoscopy in UC patients with normal controls (NC). This approach aims to uncover novel molecular insights and potential therapeutic targets, providing a deeper understanding of UC pathogenesis.

To elucidate the gene expression changes in UC, we obtained RNAseq data from 15 NCs, 15 colonic tissue samples from UCI, and 15 from UCA (Fig. S1 and Table S1). Differentially expressed gene (DEG) analysis revealed that the number of DEGs in UCA was substantially higher than those in UCI or NC (Fig. S2A and Table S2) and DEGs in UCA versus NC and those in UCA versus UCI shared more common genes than other comparisons (Fig. S2B). These findings highlight the distinct transcriptomic profiles of UCA compared with UCI and NC. To confirm the distinct expression in UCA, we performed hierarchical clustering on combined DEGs, revealing that DEGs can be clustered into four gene sets (Fig. 1A and Table S3). Three gene groups (A, B, and C) were commonly repressed in UCA. The genes in Group A were highly expressed both in NC and UCI, the genes in Group B were highly expressed only in UCI, and the genes in Group C were highly expressed only in NC However, group D showed a distinct up-regulation in UCA. The enriched gene ontology (GO) and signaling pathway terms associated with DEG group D were also distinct from the terms associated with the other groups. The terms associated with group D included inflammatory responses, signaling pathways mediated by cytokine, oncostatin M, and interleukin-1 (Fig. S2C). On the other hand, the other DEG groups were mainly associated with metabolic processes and homeostasis.

**Figure 1:**
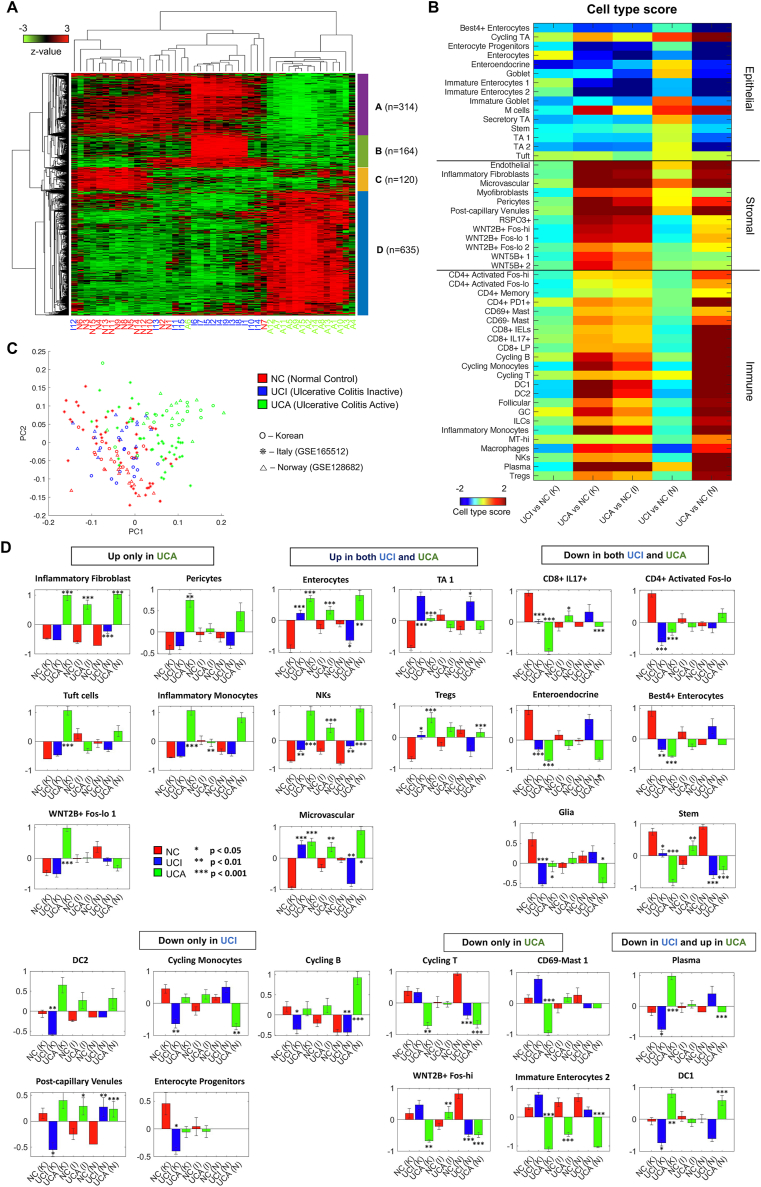
Integrative analysis using bulk and single-cell RNA sequencing revealed inflammatory transcriptomic signatures in ulcerative colitis patients. **(A)** The heatmap of the differentially expressed genes sorted by two-way hierarchical clustering. The differentially expressed genes were clustered into four groups. **(B)** The principal component analysis plot of the three datasets from Korean (our study), Italian, and Norwegian studies. **(C)** A heatmap of the 50 cell type scores for each comparison of bulk RNA sequencing data of the three datasets. The cell type scores based on single-cell RNA sequencing suggest the putative cell type contributions in the bulk RNA sequencing data. **(D)** A total of 27 cell types were significantly altered in different patient types of the three datasets. Deconvolution of RNA sequencing reveals altered cell types in active inflammatory colonic tissue (UCA). The K, I, and N in the parenthesis represent Korean, Italian, and Norwegian datasets, respectively. UCI, macroscopically inactive inflammatory colonic tissue; NC, normal control.

To validate expression profiles specific to UC in our dataset, we utilized two public bulk RNAseq data (Supplementary Methods), providing that the DEGs in UCI versus NC were not explicitly distinguished in the two datasets (Fig. S3A). However, the DEGs in UCA versus NC or UCI displayed consistent expression patterns in the two datasets (Fig. S3B, C). These results indicate that the UCA-associated expression patterns were consistently found in the independent datasets of other ethnicities, but the UCI-specific expression patterns obtained from our dataset could be dataset- or ethnicity-specific. The distinct expression patterns in UCA from three datasets were also confirmed using principal component analysis (Fig. 1B). On the other hand, the UCI patients in Korean and Norwegian datasets were not distinguishable from NC patients in PC1 and PC2 space.

The expression profile in bulk RNAseq can be modeled by a linear combination of many single cells' expression profiles. To infer the cell types in which the linear combination from our bulk RNAseq data was combined, we utilized publicly available single-cell RNA sequencing (scRNAseq) data.^5^ These data encompassed three main cell types: epithelial cells, immune cells, and fibroblasts (Fig. S4). We calculated the cell type scores for each gene and each log ratio within three groups of bulk RNAseq data, including our dataset, based on the discrete coefficient values of marker genes of 50 cell types in scRNAseq data (Fig. 1C). The heatmap demonstrated that the overall contributions of immune and stromal cell types were elevated whereas those of epithelial cell types were reduced by UCA-specific expression profile for all three datasets (Fig. 1C). On the other hand, the UCI-specific expression profiles displayed inconsistent cell type scores for Korean and Norwegian datasets. Specifically, the top marker genes for bestrophin-4-positive (Best4^+^) enterocytes and enterocyte progenitors showed clear down-regulation in UCA whereas those for endothelial cells, inflammatory fibroblasts, and inflammatory monocytes showed clear up-regulation in UCA for all three datasets (Fig. S5–7). Among the epithelial cells, M cell markers displayed clear up-regulation in UCA for the Korean and Norwegian datasets, but the up-regulation was not explicit in the Italian dataset (Fig. S5). In sum, the overall cell type contributions were similarly displayed in all three datasets, but a few cell types showed slightly different contributions in the Italian dataset. These overall patterns including up-regulation of M cells, inflammatory fibroblasts, and inflammatory monocytes, and down-regulation of Best4^+^ enterocytes in UCA are consistent with the cell type composition results in the scRNAseq study.^5^

To identify critical markers for the cell-type-specific expression patterns, we examined the genes expressed in M cells, inflammatory monocytes, and inflammatory fibroblasts among the common DEGs of two DEG groups (up-regulated in UCA versus NC or UCI) and Best4^+^ enterocytes among the common DEGs of two DEG groups (down-regulated in UCA versus NC or UCI). Figure S8A shows the expression level of the DEGs from RNAseq data in scRNAseq data, which displayed the cell type origin of the DEGs. For instance, PDPN (podoplanin), CXCL1 (C-X-C motif chemokine ligand 1), CHI3L1 (chitinase 3 like 1), and MMP1 (matrix metallopeptidase 1) may originate from inflammatory fibroblasts, while SERPINA1 (serpin family A member 1), S100A8 (S100 calcium-binding protein A8), and S100A9 (S100 calcium-binding protein A9) may originate from inflammatory monocytes among DEGs up-regulated in UCA. Similarly, SOCS1 (suppressor of cytokine signaling 1) and CXCL3 (C-X-C motif chemokine ligand 3) can originate from M cells, and SAA1 (serum amyloid A1), SLC6A14 (solute carrier family 6 member 14), and DUOXA2 (dual oxidase maturation factor 2) can originate from the enterocyte sub-cluster 2 (Fig. S8B). The enterocyte sub-cluster 2 is characterized by high expression of DEGs up-regulated in UCA versus NC or UCI (Fig. S8C). The down-regulated DEGs, GUCA2A (guanylate cyclase activator 2A), BEST4, and OTOP2 (otopetrin 2), may be derived from BEST4^+^ enterocytes. This UCA-specific differential expression of those markers was validated using real-time PCR (Fig. S8D).

To address the marker gene ambiguity in the analysis of cell type contributions in our bulk RNAseq dataset (Fig. 1C), we deconvolved our RNAseq dataset based on the scRNA-seq data (Supplementary Methods). In concordance with the cell type score analysis, the overall cell type composition showed a distinct pattern in UCA, and immune and stromal cells were enriched in UCA compared with UCI and NC (Fig. S9A). This pattern was also found in the other datasets (Fig. S9B, C). Based on the statistical tests, we identified six groups of altered cell types (Fig. 1D). We confirmed the enrichment of inflammatory fibroblasts, enterocytes, microvascular cells and natural killer cells and depletion of immature enterocyte 2 cells in UCA from the Italian and Norwegian datasets (Fig. 1D). However, some of the cell type enrichment or depletion were demonstrated for one dataset or no agreement for both datasets such as tuft cells and inflammatory monocytes. The hierarchical clustering based on the cell type compositions provides four cell-type groups, including two groups showing clear separation by patient types (Fig. S10A), with enriched or depleted in UCA. In the Italian and Norwegian datasets, the hierarchical clustering also resulted in a group enriched in UCA associated with inflammatory fibroblasts, inflammatory monocytes, natural killer cells, and plasma cells. However, other cell types were clustered in different ways in the three datasets (Fig. S10B, C).

## Ethics declaration

This study was approved by the Ewha Womans University Medical Center institutional review board (IRB number SEUMC 2020-12-017)) and colonoscopy biopsies were conducted after obtaining informed consent from participants.

## Funding

This work was supported by the Basic Science Research Program through the 10.13039/501100003725National Research Foundation of Korea (NRF) funded by the 10.13039/501100002701Ministry of Education of the Republic of Korea (No. 2020R1I1A1A01073545 to E.M.S., 2020R1A2C1010786 to C.M.M., 2021R1A6A1A10044154 to J.K).

## Author contributions

**Eun Mi Song:** Data curation, Investigation, Validation, Visualization, Writing – original draft, Writing – review & editing. **Jahanzeb Saqib:** Data curation, Investigation, Software, Visualization, Writing – original draft. **Yang Hee Joo:** Data curation, Investigation, Validation, Writing – original draft. **Zehra Ramsha:** Data curation, Investigation, Writing – original draft. **Chang Mo Moon:** Conceptualization, Data curation, Funding acquisition, Project administration. **Sung-Ae Jung:** Conceptualization, Funding acquisition, Project administration, Supervision. **Junil Kim:** Data curation, Funding acquisition, Investigation, Methodology, Project administration, Supervision, Visualization, Writing – original draft, Writing – review & editing.

## Data availability

The RNAseq data can be downloaded from the Gene Expression Omnibus database with accession number GSE 243625.

## Conflict of interests

The authors declared no conflict of interests.
