# Supplementary material for: Inflammatory transcriptomic signatures and cell type compositions in inflamed and non-inflamed colonic mucosa of ulcerative colitis
Source: Genes Dis. 2024 Oct 30;12(3):101447. doi: 10.1016/j.gendis.2024.101447 (PMC11804533; doi:10.1016/j.gendis.2024.101447)
Supplement: Multimedia component 1 [file mmc1.docx]

**Inflammatory transcriptomic signatures and cell type compositions in inflamed and non-inflamed colonic mucosa of ulcerative colitis**

**Supplementary Material**

Eun Mi Song^a*^, Jahanzeb Saqib^b*^, Yang Hee Joo^a^, Zehra Ramsha^b^, Chang Mo Moon^a^, Sung-Ae Jung^a^**^†^**, Junil Kim^b^**^†^**

*^1^Departments of Internal Medicine, College of Medicine, Ewha Womans University, Seoul 07804, Republic of Korea*

*^2^School of Systems Biomedical Science, Soongsil University, 369 Sangdo-Ro, Dongjak-Gu, Seoul 06978, Republic of Korea*

*These authors contributed equally as co-first authors.

**^†^**These authors contributed equally as co-corresponding authors.

**Correspondences:**

Junil Kim, PhD; School of Systems Biomedical Science, Soongsil University, 369 Sangdo-Ro, Dongjak-Gu, Seoul 06978, Republic of Korea; Tel.: +82-2-820-0452; E-mail: junilkim@ssu.ac.kr

Sung-Ae Jung, MD, PhD; Departments of Internal Medicine, College of Medicine, Ewha Womans University, Seoul, Republic of Korea; Tel.: +82-2-6986-1620; E-mail: jassa@ewha.ac.kr

**Supplementary Methods**

**Sample preparation from patients with UC**

The study focused on patients undergoing treatment for UC at Ewha Woman University Seoul Hospital between 2020 and 2021. The participants were selected from the Ewha IBD registry, which had been systematically collecting clinical data, colon biopsy samples, blood samples, and stool samples since 2019 under the IRB number SEUMC 2019-04-019. Specifically, individuals with UC who underwent colonoscopy during the study period and exhibited both actively inflamed and non-inflamed mucosa in a single examination were recruited for the study in a prospective manner. Following approval from the Institutional Review Board (IRB number SEUMC 2020-12-017) and after obtaining informed consent from participants, colon biopsies were conducted. Biopsy samples were obtained from the colonic mucosa in areas both with active inflammation and without inflammation during the colonoscopy. Each biopsy sample, both from inflamed and non-inflamed tissue, was individually preserved using the RNAlater® (Invitrogen, MA, USA) stabilizing agent at a temperature of -80 °C until analysis. Furthermore, within the same study period, healthy individuals without abnormal colonoscopy findings were recruited as normal controls during colonoscopy screenings. Normal colonic tissue samples were also collected from these healthy control participants and stored using the same RNAlater® (Invitrogen, MA, USA) preservation method. The diagnosis of UC was established based on internationally accepted diagnostic criteria, which involved a comprehensive assessment encompassing clinical, endoscopic, histologic, and radiologic examinations^1^. A total of 45 samples were gathered and categorized into three groups: healthy normal controls (NC, n=15), colonic tissue without active inflammation in patients with UC (UC-inactive, UCI, n=15), and colonic tissue with active inflammation in patients with UC (UC-active, UCA, n=15).

**RNAseq**

RNA quality was assessed using the RIN (RNA Integrity Number) algorithm on the Agilent 2100 bioanalyzer. The cDNA library was constructed with 1.0 μg of total RNA using NEBNext Ultra II directional RNA library preparation kit for Illumina sequencer. Then, 100 bp paired-end sequencing reads were obtained using Illumina NovaSeq 6000.

The RNAseq reads were trimmed by BBDuk^2^ with quality-trim=20 option. The trimmed reads were mapped to the human reference genome GRCh37 using TopHat^3^. Based on the mapping result, we computed Fragments Per Kilobase of transcript per Million mapped reads (FPKM) using Cufflinks^4^. Differentially expressed genes (DEGs) were obtained by comparing the three groups (UCA, UCI, and NC) using EdgeR^5^. Genes with a p-value<0.01 and log2fold change>1 were selected as DEGs. The union of DEGs were clustered using hierarchical clustering and principal component analysis (PC) implemented in MATLAB R2020a. The enriched terms associated with clustered genes were obtained using Enrichr software^6^.

**Public single-cell RNAseq data and bulk RNAseq data analysis**

We obtained a publicly available single-cell RNAseq (scRNAseq) dataset^7^ containing data from 18 patients with UC and 12 NCs. In addition, to validate expression profiles specific to UC in our own dataset, we utilized two additional publicly available bulk RNAseq data, consisting of 46 NC and 40 UCA patients in an Italian study (GSE165512, unpublished) and 16 NC, 14 UCI, and 14 UCA patients in a Norwegian study (GSE128682)^8^. To infer cell type scores for the bulk RNA sequencing data, we utilized the marker genes associated with 50 different cell types provided in the scRNAseq study, The cell type score $C_{i}$ for cell type *i* is defined as follows:

$C_{i}\equiv\sum_{j}^{M} \frac{d_{j}\left( i \right)\times l_{j}}{M}$ (1)

where *M* represents the total number of marker genes for cell type *i*, $d_{j}\left( i \right)$ is the discrete differential expression coefficient of gene *j* for cell type *i*, $l_{j}$ is the log ratio of gene *j* in the bulk RNA sequencing data. The discrete differential expression coefficient $d_{j}\left( i \right)$ indicates how indicative a gene is for a specific cell type and is based on the original scRNAseq study. The cell type score represents the contribution of cell type expression profiles in the transcriptomic changes of bulk RNA sequencing.

**Deconvolution of RNAseq using scRNAseq data**

To deconvolve the bulk RNAseq into cell type proportions, we employed the CIBERSORTx algorithm^9^. The transcriptomic signature was calculated based on the scRNAseq data. To reduce the effect of cross-platform variance on the results, we chose single-cell mode, termed "S-mode". After running CIBERSORTx, we estimated the proportions of 51 cell types in every sample. The significantly altered cell type compositions between the three groups were obtained by two-sided Student’s t-tests with a p-value<0.05.

**Real time polymerase chain reaction**

RNA extraction and reverse transcription were conducted following the protocols provided by the manufacturer. Subsequently, the Quantstudio 3 real-time polymerase chain reaction (PCR) system (Applied Biosystems, Waltham, Massachusetts, USA) was used for analysis using 0.1 μg of synthesized cDNA as a template for the 2X Power SYBR Green PCR Master mix (Applied Biosystems). Specific primer set for each gene from Macrogen (Seoul, Korea) was employed (detailed in Table S4). Each PCR was performed after 10 min of pre-denaturation at 95 °C, 15 s at 95 °C, and 1 min at 60 °C; this was repeated 40 times. After PCR was completed, a melting curve was drawn to check the accuracy of gene amplification. For the internal compensation of gene expression level, the housekeeping gene glyceraldehyde-3-phosphate dehydrogenase (GAPDH) was also used, and relative gene expression levels were presented as 2-^ΔΔ^Ct values.

**Supplementary Figures**

**Figure S1. A cartoon of colon tissue for sample preparation of RNAseq.**

**Figure S2. DEGs found in UC samples using RNAseq.** (**A**) Volcano plots show that the number of DEGs in UCA versus UCI or NC is much higher than that in UCI versus NC. (**B**) A Venn diagram illustrating a significant overlap between the DEGs in UCA versus NC and DEGs in UCA versus UCI. (**C**) KEGG pathways and GO terms associated with four groups of DEGs. (**D**) BioPlanet pathways associated with gene group D.

**Figure S3. The expression patterns of the DEGs in three different datasets.** (**A-C**) Heatmaps of the DEGs for UCI vs NC (**A**), UCA vs NC (**B**) and UCA vs UCI (**C**) of the three datasets from Korean (our study), Italian, and Norwegian studies. The z-values were calculated within each dataset.

**Figure S4. Three t-SNEs depict scRNAseq data, each corresponding to one of three cell types: epithelial cells, immune cells, stromal cells.** Within these plots, the names of 51 cell types are positioned atop the center of their cell type’s location.

**Figure S5. Log ratio of top 20 cell type marker genes for 15 epithelial cell types in bulk RNA sequencing data.**

**Figure S6. Log ratio of top 20 cell type marker genes for 12 stromal cell types in bulk RNA sequencing data.**

**Figure S7. Log ratio of top 20 cell type marker genes for 23 immune cell types in bulk RNA sequencing data.**

**Figure S8. The origin of cell types of the DEGs from bulk RNAseq.** (**A**) A dot plot of cell-type-specific marker genes among the DEGs upregulated or downregulated in UCA in scRNAseq data. (**B**) Sub-clustering of enterocytes yielded three clusters. (**C**) A heatmap of three enterocyte sub-clusters demonstrates UCA-specific expression in sub-cluster 2. (**D**) Validation of the expression profile of the marker genes in bulk RNAseq using real time PCR.

**Supplementary Figure S9. Deconvolution of RNAseq reveals altered cell type compositions in UCA from three different datasets.** (**A**) Korean (**B**) Italian (**C**) Norwegian datasets.

**Figure S10. Hierarchical clustering based on the cell type compositions in three datasets of UC.** (**A**) Korean (**B**) Italian (**C**) Norwegian datasets. Red box in subfigure A represent two distinct groups of cell types displaying enrichment and depletion of those cell types in UCA. We underlined examples of distinct cell types found in the Korean dataset.

**Supplementary Discussion**

In this study, we investigated differences in the transcriptomes of colonic mucosa samples with active and inactive inflammation from patients with UC (UCA and UCI), and NCs using RNAseq. Standard transcriptomics analysis revealed distinct transcriptomic changes in UCA mucosa, primarily associated with inflammation-associated genes. Furthermore, by incorporating publicly available scRNAseq data, we found that the marker genes of three inflammation-related cell types were upregulated in UCA, including M cell, inflammatory monocytes, and inflammatory fibroblasts. Conversely, the marker genes for some other cell types such as BEST4+ enterocytes and enterocyte progenitors were downregulated in UCA. In our deconvolution analysis of the RNAseq data using CIBERSORTx^9^, we predicted significant enrichment of NK cells, inflammatory monocytes, Tuft cells, inflammatory fibroblasts, WNT2B+Fos-lo1, and pericytes in the UCA samples. Many of these transcriptomic patterns were consistent with the previous studies from Italian and Norwegian samples. A strength of our study lies in its extensive analysis of the distinctive transcriptome signature of treatment-unresponsive active colonic mucosal lesions, particularly in Asian patients with UC, through an integrative transcriptome analysis.

Using bulk RNAseq, the identified transcriptomic changes in UCA were primarily associated with inflammatory responses, cytokine signaling pathways, and granulocyte/neutrophil chemotaxis (Fig. S1C). These findings align with previous studies on patients with IBD using RNAseq, indicating an upregulation of genes associated with pro-inflammatory cytokines and innate immunity in UC with active inflammation^7,8,10–13^. In our study, genes specific to UCA were enriched in BioPlanet pathways, including “Oncostatin M,” “interleukin-1 regulation of extracellular matrix,” “TNFα effects on cytokine activity, cell motility and apoptosis,” “TGFβ regulation of extracellular matrix,” and “leptin influence on immune response.” Our findings were consistent with recently published transcriptomic analyses involving a large cohort of patients with IBD from the United States^14^. This result suggests that these pathways are relevant not only to Western patients with UC but also to Asian patients with UC, demonstrating the consistency of these findings across diverse populations. Additionally, our results suggest potential novel therapeutic candidates, such as Oncostatin M, interleukin-1, and leptin in IBD treatment.

Bulk RNAseq measures RNA from mixed cells, making it challenging to identify cell type-specific gene expression. By leveraging the power of scRNAseq, we addressed this limitation. Using a publicly available scRNAseq dataset^7^, we analyzed our RNAseq data using two different methods: gene-based and sample-based. In the gene-based approach, we assigned cell type origins of 18 DEGs obtained from RNAseq (Figs. 1D and S4-S6). Especially, we confirmed the upregulation of SAA1, SLC6A14, and DUOXA2 in UCA using real time PCR. These three genes were identified using single cell analysis of a subcluster of enterocytes, which was highlighted in the original scRNAseq study^7^. Recent studies conducted in the United States identified SAA1 and DUOXA2 as significant indicators for distinguishing mucosal lesions with active inflammation in UC, suggesting a potential mechanism involving the interaction between T cell lineages and microbial communities^15,16^. In another study, SLC6A14 was proposed as a potential biomarker for assessing UC activity and gene that responds differentially to gut microbes^17^. Taken together, our results indicate a potential link between the persistent active mucosal inflammation in UC, even after treatment, and a distinct expansion within enterocyte sub-cluster 2, which is triggered by a crosstalk between gut microbiota and innate immunity.

In the sample-based approach, we deconvolved our RNAseq data, revealing cell type compositions for each sample using CIBERSORTx^9^, a deconvolution tool with superior performance^18^. The deconvolution into 51 cell types revealed that inflammatory monocytes, fibroblasts, and NK cells are highly enriched in UCA (Figs. 1E and S8), supporting the gene-based integrative analysis (Figs. 1D and S4-S6). UC involves complex interactions between immune and stromal cells, mediated by cytokines^19^. Our analysis identified key cell types, such as inflammatory monocytes and fibroblasts, as potential drivers of prolonged inflammation. Enriched NK cells also play a crucial role by activating immune cells and enhancing antigen presentation, contributing to active inflammation in UC^20^. The cell type compositions of these cell types were mostly consistent in the other independent RNAseq datasets obtained from Italian and Norwegian samples.

Additionally, pericytes, tuft cells, and WNT2B+Fos-lo1 fibroblasts were also enriched in UCA. Pericytes, in particular, are known to drive angiogenesis, making them potential therapeutic targets in various diseases, including IBD^21^. The enrichment of pericytes in UCA, consistent with findings from a study conducted in Italy^22^, highlights their role in micro-vessel angiogenesis across the colonic wall. Our findings reinforce the critical role of angiogenesis in UC onset and progression. Moreover, enriched pericytes, known as a source of extracellular matrix components that produce myofibroblast, suggest inflammation-induced fibrosis and tissue remodeling in UCA^23^.

Tuft cells are known to play a role in immune responses within the intestinal epithelial barrier^24^. However, the precise involvement of tuft cells in gastrointestinal diseases remains incompletely characterized, and conflicting results have been reported. Kjærgaard *et al*. reported a decrease in the number of colonic tuft cells is decreased in UCI (histological remission) samples^25^. In contrast, another study observed an increase in tuft cell numbers in the human stomach during inflammation^26^. In our analysis of Korean datasets, we predicted an increase in tuft cells in UCA samples, although this finding was not significant in the Italian and Norwegian datasets. The observed discrepancies may be attributed to various factors, including differences in endoscopy procedures, RNA sequencing techniques or the ethnicity of the study populations. This underscores the need for further investigation into the role of tuft cells in gastrointestinal diseases in future study.

Our study also evaluated the transcriptomic characteristics of non-inflamed colonic mucosa in UC shedding light on early-stage mechanisms and treatment effects. Fenton *et al*.^8^ identified a small number of specific DEGs that were associated with barrier function (MUC12, MUC17), while Söderman *et al*.^10^ revealed 52 genes associated with tissue homeostasis, remodeling, and immune response in the colonic mucosa of UC in remission. In our dataset, we identified 164 upregulated genes specific to UCI (gene group B in Fig. 1B) and 120 downregulated genes specific to UCI (gene group C in Fig. 1B), which were mainly associated with tissue homeostasis, metabolic processes, and developmental processes, aligning with the previous studies. Interestingly, cell-type analysis revealed that the composition of Best4+ enterocytes decreased in the order of NC, UCI, and UCA. The maintenance of these cells, crucial for fluid and electrolyte balance as well as gut homeostasis, in UCI lesions may represent a potential mechanism against inflammation in IBD pathogenesis. The depletion of Best4+ enterocytes in UCA was also highlighted in the scRNAseq study. However, the decreasing pattern of Best4+ enterocytes was not found in the deconvolution analysis from the Italian and Norwegian datasets (Fig. 1E).

Single cell transcriptomics enables the study of cell type-specific alterations in many diseases that cannot be found in bulk RNAseq analysis. However, bulk RNAseq still has potential since single cell sequencing for large population is limited due to the high cost. Our study showed that the cell type composition can be successfully studied by integrating bulk RNAseq with cell type expression profiles based on single cell RNAseq. This suggests that a study design of the combination of single cell and bulk RNAseq can be an effective strategy for acquiring both cell type profiling and large population.

In conclusion, our work provides new insights into the transcriptomic characteristics of colonic mucosa, both with and without active inflammation despite treatment, in Asian patients with UC. We have identified significant changes in not only gene expression but also cell type proportion associated with persistent inflammation, which can be a valuable resource for the development of UC treatment strategies.

**References**

1. Gomollón F, Dignass A, Annese V, et al. 3rd European evidence-based consensus on the diagnosis and management of Crohn’s disease 2016: Part 1: Diagnosis and medical management. *J Crohn’s Colitis*. 2017;11(1):3-25.

2. Bushnell B, Rood J, Singer E. BBMerge – Accurate paired shotgun read merging via overlap. *PLoS One*. 2017;12(10):e0185056.

3. Kim D, Pertea G, Trapnell C, Pimentel H, Kelley R, Salzberg SL. TopHat2: Accurate alignment of transcriptomes in the presence of insertions, deletions and gene fusions. *Genome Biol*. 2013;14(4):1-13.

4. Trapnell C, Williams BA, Pertea G, et al. Transcript assembly and quantification by RNA-Seq reveals unannotated transcripts and isoform switching during cell differentiation. *Nat Biotechnol*. 2010;28(5):511-515.

5. Robinson MD, McCarthy DJ, Smyth GK. edgeR: a Bioconductor package for differential expression analysis of digital gene expression data. *Bioinformatics*. 2010;26(1):139-140.

6. Kuleshov M V., Jones MR, Rouillard AD, et al. Enrichr: a comprehensive gene set enrichment analysis web server 2016 update. *Nucleic Acids Res*. 2016;44(W1):W90-W97.

7. Smillie CS, Biton M, Ordovas-Montanes J, et al. Intra- and inter-cellular rewiring of the human colon during ulcerative colitis. *Cell*. 2019;178(3):714-730.e22.

8. Fenton CG, Taman H, Florholmen J, Sørbye SW, Paulssen RH. Transcriptional signatures that define ulcerative colitis in remission. *Inflamm Bowel Dis*. 2021;27(1):94-105.

9. Newman AM, Steen CB, Liu CL, et al. Determining cell type abundance and expression from bulk tissues with digital cytometry. *Nat Biotechnol 2019 377*. 2019;37(7):773-782.

10. Söderman J, Berglind L, Almer S. Inverse and concordant mucosal pathway gene expressions in inflamed and non‐inflamed ulcerative colitis patients: Potential relevance to aetiology and pathogenesis. *Int J Mol Sci*. 2022;23(13):6944.

11. Kinchen J, Chen HH, Parikh K, et al. Structural Remodeling of the Human Colonic Mesenchyme in Inflammatory Bowel Disease. *Cell*. 2018;175(2):372-386.e17.

12. Eshelman MA, Jeganathan NA, Schieffer KM, et al. Elevated Colonic Mucin Expression Correlates with Extended Time to Surgery for Ulcerative Colitis Patients. *J Gastrointest Liver Dis*. 2019;28(4):405-413.

13. Mo A, Krishnakumar C, Arafat D, et al. African Ancestry Proportion Influences Ileal Gene Expression in Inflammatory Bowel Disease. *Cell Mol Gastroenterol Hepatol*. 2020;10(1):203-205.

14. Argmann C, Hou R, Ungaro RC, et al. Biopsy and blood-based molecular biomarker of inflammation in IBD. *Gut*. 2023;72(7):1271-1287.

15. Tang MS, Bowcutt R, Leung JM, et al. Integrated analysis of biopsies from inflammatory bowel disease patients Identifies SAA1 as a link between mucosal microbes with TH17 and TH22 cells. *Inflamm Bowel Dis*. 2017;23(9):1544-1554.

16. MacFie TS, Poulsom R, Parker A, et al. DUOX2 and DUOXA2 form the predominant enzyme system capable of producing the reactive oxygen species H2O2 in active ulcerative colitis and are modulated by 5-aminosalicylic acid. *Inflamm Bowel Dis*. 2014;20(3):514-524.

17. Chen Y, Yan W, Chen Y, et al. SLC6A14 facilitates epithelial cell ferroptosis via the C/EBPβ-PAK6 axis in ulcerative colitis. *Cell Mol Life Sci*. 2022;79(11):1-15.

18. Sutton GJ, Poppe D, Simmons RK, et al. Comprehensive evaluation of deconvolution methods for human brain gene expression. *Nat Commun 2022 131*. 2022;13(1):1-18.

19. Filer A, Pitzalis C, Buckley CD. Targeting the stromal microenvironment in chronic inflammation. *Curr Opin Pharmacol*. 2006;6(4):393-400.

20. Zaiatz Bittencourt V, Jones F, Tosetto M, Doherty GA, Ryan EJ. Dysregulation of metabolic pathways in circulating natural killer cells isolated from inflammatory bowel disease patients. *J Crohn’s Colitis*. 2021;15(8):1316-1325.

21. Gerhardt H, Betsholtz C. Endothelial-pericyte interactions in angiogenesis. *Cell Tissue Res*. 2003;314(1):15-23.

22. Ippolito C, Colucci R, Segnani C, et al. Fibrotic and vascular remodelling of colonic wall in patients with active ulcerative colitis. *J Crohn’s Colitis*. 2016;10(10):1194-1204.

23. Li C, Kuemmerle JF. The fate of myofibroblasts during the development of fibrosis in Crohn’s disease. *J Dig Dis*. 2020;21(6):326-331.

24. Hendel SK, Kellermann L, Hausmann A, Bindslev N, Jensen KB, Nielsen OH. Tuft Cells and Their Role in Intestinal Diseases. *Front Immunol*. 2022;13:822867.

25. Kjærgaard S, Jensen TSR, Feddersen UR, et al. Decreased number of colonic tuft cells in quiescent ulcerative colitis patients. *Eur J Gastroenterol Hepatol*. 2021;33(6):817-824. doi:10.1097/MEG.0000000000001959

26. Saqui-Salces M, Keeley TM, Grosse AS, et al. Gastric tuft cells express DCLK1 and are expanded in hyperplasia. *Histochem Cell Biol*. 2011;136(2):191-204. doi:10.1007/S00418-011-0831-1/TABLES/2
